# Plants of the Genus *Zingiber* as a Source of Bioactive Phytochemicals: From Tradition to Pharmacy

**DOI:** 10.3390/molecules22122145

**Published:** 2017-12-04

**Authors:** Mehdi Sharifi-Rad, Elena Maria Varoni, Bahare Salehi, Javad Sharifi-Rad, Karl R. Matthews, Seyed Abdulmajid Ayatollahi, Farzad Kobarfard, Salam A. Ibrahim, Dima Mnayer, Zainul Amiruddin Zakaria, Majid Sharifi-Rad, Zubaida Yousaf, Marcello Iriti, Adriana Basile, Daniela Rigano

**Affiliations:** 1 Department of Medical Parasitology, Zabol University of Medical Sciences, Zabol 61663335, Iran; mehdi_sharifirad@yahoo.com; 2 Department of Biomedical, Surgical and Dental Sciences, Milan State University, 20133 Milan, Italy; elena.varoni@unimi.it; 3 Zabol Medicinal Plants Research Center, Zabol University of Medical Sciences, Zabol 61615585, Iran; 4 Phytochemistry Research Center, Shahid Beheshti University of Medical Sciences, Tehran 11369, Iran; majid_ayatollahi@yahoo.com (S.A.A.); farzadkf@yahoo.com (F.K.); 5 Department of Chemistry, Richardson College for the Environmental Science Complex, The University of Winnipeg, 599 Portage Avenue, Winnipeg, MB R3B 2G3, Canada; 6 Department of Food Science, Rutgers University, New Brunswick, NJ 08901, USA; bijan@sebs.rutgers.edu; 7 Department of Pharmacognosy, School of Pharmacy, Shahid Beheshti University of Medical Sciences, Tehran 11369, Iran; 8 Department of Medicinal Chemistry, School of Pharmacy, Shahid Beheshti University of Medical Sciences, Tehran 11369, Iran; 9 Food Microbiology and Biotechnology Laboratory, 171 Carver Hall, College of Agriculture and Environmental Sciences, North Carolina A & T State University, Greensboro, NC 27411, USA; ibrah001@ncat.edu; 10 Faculty of Agricultural Engineering and Veterinary Medicine, Lebanese University, Dekwaneh, Beirut 6573, Lebanon; d_mnayer@yahoo.fr; 11 Department of Biomedical Sciences, Faculty of Medicine and Health Sciences, Universiti Putra Malaysia, Serdang 43400, Selangor, Malaysia; dr_zaz@yahoo.com; 12 Integrative Pharmacogenomics Institute (iPROMISE), Level 7, FF3 Building, Universiti Teknologi MARA, Puncak Alam 42300, Selangor, Malaysia; 13 Department of Range and Watershed Management, Faculty of Natural Resources, University of Zabol, Zabol 98615538, Iran; majid.sharifirad@gmail.com; 14 Department of Botany, Lahore College for Women University, Jail Road Lahore 54000, Pakistan; mussabuswaeshal@hotmail.com; 15 Department of Agricultural and Environmental Sciences, Milan State University, 20133 Milan, Italy; 16 Department of Biological Sciences—Plant Biology Section, University of Naples “Federico II”, 80126 Naples, Italy; adbasile@unina.it; 17 Department of Pharmacy, University of Naples Federico II, via D. Montesano 49, 80131 Naples, Italy

**Keywords:** *Zingiber*, ginger, essential oil, rhizome, herbal remedies, traditional healing systems, food preservatives

## Abstract

Plants of the genus *Zingiber* (Family Zingiberaceae) are widely used throughout the world as food and medicinal plants. They represent very popular herbal remedies in various traditional healing systems; in particular, rhizome of *Zingiber* spp. plants has a long history of ethnobotanical uses because of a plethora of curative properties. Antimicrobial activity of rhizome essential oil has been extensively confirmed in vitro and attributed to its chemical components, mainly consisting of monoterpene and sesquiterpene hydrocarbons such as α-zingiberene, ar-curcumene, β-bisabolene and β-sesquiphellandrene. In addition, gingerols have been identified as the major active components in the fresh rhizome, whereas shogaols, dehydrated gingerol derivatives, are the predominant pungent constituents in dried rhizome. *Zingiber* spp. may thus represent a promising and innovative source of natural alternatives to chemical food preservatives. This approach would meet the increasing concern of consumers aware of the potential health risks associated with the conventional antimicrobial agents in food. This narrative review aims at providing a literature overview on *Zingiber* spp. plants, their cultivation, traditional uses, phytochemical constituents and biological activities.

## 1. Introduction

Today’s consumers are becoming more aware of the link between diet and health. For instance, the Mediterranean diet possesses a plethora of bioactive phytochemicals, e.g., polyphenols and melatonin [1,2], which can contribute to its beneficial effects against chronic-degenerative disorders, including cancer, cardiovascular diseases and diabetes [3,4,5]. This trend is leading to increasing demand for the use of natural ingredients as food supplements and preservatives. In addition, an increasing number of microorganisms that are not only antibiotic resistant, but are more tolerant to existing preservative techniques is of worldwide concern [6]. Plant derived extracts that have biological activities, such as antimicrobial, antidiabetic or antihypertensive effects, have the potential to fulfill the need for safe natural preservatives [7,8,9,10,11,12]. Therefore, much emphasis has been put on the investigation of plant derived natural sources of antimicrobials, compounds that could potentially replace chemical preservatives and synthetic antimicrobial ingredients [13,14,15,16,17].

Herbs and spices, which are an important part of the human diet, have been used for thousands of years to enhance the flavor, color and aroma of food. In addition to boosting flavor, herbs and spices are also known for their preservative, antioxidant, antimicrobial and other medicinal properties [8,16,18]. The genus *Zingiber*, belonging to the family Zingiberaceae, comprises about 85 species of herbs mostly grown in Asia, Central, South America and Africa [19]. Although different members of this genus are somewhat similar in morphology, they differ widely in their pharmacological and therapeutic properties [20].

The most popular spice, ginger, comes from the underground stems or rhizomes of the plant *Zingiber officinale* Roscoe. It has been widely reported in literature that ginger is consumed worldwide as a spice and flavoring agent and is attributed to having many medicinal properties [19,21,22,23].

The essential oil (EO) from *Zingiber* rhizome is pale yellow to light-amber, contains both aromatic and pungent compounds and can be extracted with yields ranging from 1.5–3.0%, depending on the quality of the crop [24].

Phytochemical investigation of the rhizomes of several *Zingiber* spp. has revealed the presence of bioactive compounds such as gingerols, shogaols, diarylheptanoids, phenylbutenoids, flavanoids, diterpenoids and sesquiterpenoids [22]. The gingerols are identified as the major active components in the fresh rhizome of the plant. In addition, shogaols, dehydrated gingerol derivatives, are the predominant pungent constituents in dried ginger [25].

*Zingiber* plants possess various pharmacological and physiological effects and are common ingredients in traditional medicines. The rhizomes have been shown to be effective in the treatment of several medical conditions including stomach problems, nausea, vomiting, epilepsy, sore throat, cough, common cold, bruises, wounds, liver complaints, rheumatism, muscular pains, atherosclerosis, migraine headaches, high cholesterol, ulcers, and stomach discomfort [26]. In addition, phenolic compounds, especially the gingerols, in ginger root have been shown to have chemopreventive effects that have been associated with their antioxidant and anti-inflammatory activities [26].

The EOs of *Zingiber* rhizomes are used for preserving various foods against autoxidation and microbial spoilage because of their antioxidant and antimicrobial properties [24,27,28]. Many in vitro studies demonstrated the antimicrobial potential of *Zingiber* plant extracts against both Gram-positive (*Bacillus cereus*, *Staphylococcus aureus*) and Gram-negative (*Escherichia coli*, *Salmonella typhi*, *Pseudomonas aeruginosa*, *Klebsiella pneumonia*) bacteria [29]. The EOs also exhibited significant antifungal activity against *Candida glabrata*, *C. albicans* and *Aspergillus niger* [19]. These results suggest that EO of *Zingiber* plant could be used in the treatment of many bacterial and fungal diseases as well as in food preservation as natural preservatives [20,24]. The main aim of this paper is to provide an overview of the biological activity of the *Zingiber* spp. EOs and their components focus on the potential application of *Zingiber* plants as natural preservatives for foods.

## 2. Genus *Zingiber* Plant Cultivation

Most *Zingiber* spp. plants are aromatic, perennial herbs with the characteristics of growing horizontal or fibrous rhizomes. They are cultivated in many countries, though these plants prefer moist, tropical conditions. Ginger thrives in well drained soils like sandy loam, clay loam, red loam or lateritic loam. A friable loam with a pH of 6.0 to 6.5 rich in humus is optimal for production of *Zingiber*. The crop prefers a temperature range of 19–28 °C and a humidity of 70–90% [30]. Before cultivation, the land needs to be ploughed four to five times. Ginger is propagated by portions of rhizomes known as seed rhizomes. Preserved seed rhizomes are carefully cut into small pieces of 2.5–5.0 cm length weighing 20–25 g; each of the pieces should have one or two buds. China, Indonesia, Nigeria, Philippines, Thailand and India are the main ginger producers. Ginger reaches full maturity in 210–240 days after planting. Harvesting of ginger for vegetable purposes starts after 180 days based on the demand. As ginger is used in many different forms and high amounts are consumed in the countries where it is grown or is used to provide seeds for the next crop, it is difficult to get accurate figures of trade of *Zingiber* [31,32]. To enhance the productivity of crops, in some countries, at the time of planting, people use decomposed cattle manure or compost at 25–30 t/ha, either by broadcasting over the beds prior to planting or applying them in the pits at the time of planting. Rhizome seed generation is difficult and expensive. Therefore, people are looking for other techniques to cultivate *Zingiber* cost effectively.

A transplanting technique in ginger by using single bud sprouts (about 5 g) is recommended to produce high quality planting material with reduced cost. The technique involves raising transplants from single sprout seed rhizomes in the pro-tray and planting in the field after 30–40 days. This technique is greatly useful for reducing the quantity of rhizome seed [30]. To improve the quality, in some countries, crop rotation and intercropping techniques are also used. Ginger is usually intercropped in coconut, areca nut, coffee and orange plantations in Kerala and Karnataka [30]. Changes in soil fertility for improved growth of ginger under different quantities of pigeon pea hedgerow alley management produced a significant increase in ginger yield [33]. Another important technique is propagation by using internodal cuttings [34]. Aeroponic cultivation of ginger has also been introduced [35], as well as micropropagation and cytogenetic assessment [36].

## 3. Chemical Composition of Essential Oils Obtained from Genus *Zingiber* Plants

The genus *Zingiber* is widely used in the world for its medicinal and biological properties [37]. Among this genus, *Z. officinale* (ginger) is well known and mostly studied for its health benefits [38,39].

As previously introduced, the color of *Z. officinale* EO varies from pale yellow to light amber and the extraction yield ranges from 1.5% to 3% [40]. Different studies have documented their biological properties such as antimicrobial, antioxidant, cytotoxic, insecticidal [41,42], and anti-inflammatory effects [39] as well as food preservative characteristics [43].

**Figure 1:**
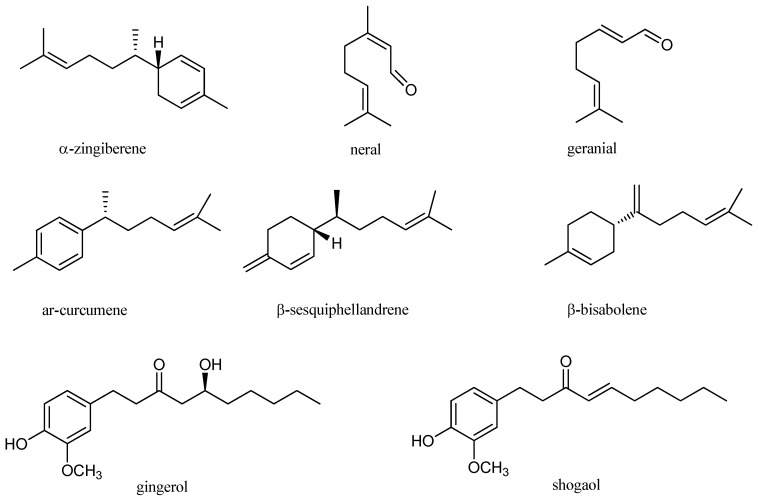
Chemical structures of major components of *Z. officinale.*

These properties have been attributed to the chemical components of *Z. officinale* EO, mainly consisting in monoterpene and sesquiterpene hydrocarbons (Figure 1) [42]. The most abundant compounds are α-zingiberene, responsible for the distinctive flavor and aroma, geranial, ar-curcumene, β-bisabolene, β-sesquiphellandrene and neral [27,44,45,46]. Other pungent constituents found in lower amount are gingerol and shogaol [47].

However, the amount and the composition of the bioactive substances may vary among different *Zingiber* species, and, according to different factors such as the extraction methods, the geographic and the growing conditions, the harvest time, etc. [46,47,48,49]. Table 1 shows the major constituents of EOs from different *Zingiber* species, the extraction methods used and their biological activities.

*Z. officinale* EO was obtained using different extraction methods including conventional hydrodistillation, microwave-assisted hydrodistillation, solvent free microwave hydrodistillation and improved solvent-free microwave extraction with three types of microwave-absorption medium. Results showed that α-zingiberene was the most abundant compound found in all EOs studied ranging from 17.4 to 25.4%, followed by ar-curcumene (14.1–16.4%), β-bisabolene (9.9–12.5%) and β-sesquiphellandrene (9.7–13.4%) [46]. These results are in accordance with those obtained from dried ginger rhizome EO, showing that the major components were α-zingiberene (29.5%) and sesquiphellandrene (18.4%) [50]. Again, α-zingiberene was reported as a major constituent (28.62%) found in the fresh rhizome EO of *Z. officinale*, followed by camphene (9.32%), ar-curcumene (9.09%) and β-phellandrene (7.97%) [45].

A comparative chemical composition was conducted on fresh and dry rhizome EOs belonging to *Z. officinale* cv. Nedumangadu. α-Zingiberene was the major compound found both in fresh and dry ginger EOs (28.6 and 30.9%, respectively). Fresh ginger EO also contained geranial (8.5%), ar-curcumene (5.6%) and β-bisabolene (5.8%), whereas ar-curcumene (11%), β-bisabolene (7.2%), β-sesquiphellandrene (6.6%) and germacrene-D (4.2%) were present in the dry ginger EO. Fresh ginger EO exhibited higher antibacterial activity due to the oxygenated compounds (29.2%), which are higher than in dry ginger EO (14.4%) [51].

Many *Z. officinale* cultivars were studied and compared for their EO composition. Seventeen cultivars from north India were studied for their chemical composition and the major components were camphene (8.49 ± 0.41%), neral (4.95 ± 0.34%), geranial (12.36 ± 0.46%), α-zingiberene (20.98 ± 2.34%) and β-sesquiphellandrene (7.96 ± 0.66%) [52].

Three sub Himalayan ginger cultivars, namely Gorubathane, Shingboi and Thingria were studied for their EO composition. Results showed that α-zingiberene (32.2%) and β-sesquiphellandrene (10.9%) were the major compounds in Gorubathane EO, whereas α-zingiberene (12.58%) and ar-curcumene (9.89%) were mostly present in Thingria EO. However, geranial (20.07%) and neral (9.44%) were the main constituents found in Shingboi EO [44].

The composition of EO may vary not only within different ginger cultivars, but also according to the parts of the plant studied, as demonstrated by Sivasothy et al. [22], who showed that the composition of EO obtained by hydrodistillation of the leaves and rhizomes of *Z. officinale* var. Rubrum Theilade were different. In fact, β-caryophyllene (31.7%) was the major compound found in the leaf oil, while rhizome oil was predominantly rich in monoterpenoids, such as camphene (14.5%), geranial (14.3%) and geranylacetate (13.7%).

Regarding the extraction methods used, water and steam distillation were used to obtain EO from Vietnamese *Z. officinale*. The main EO components obtained by water distillation were α-cucurmene (11.7%) and β-bisabolene (4.1%), while those obtained by steam distillation were ar-curcumene (12.6%), α-zingiberene (10.3%), β-bisabolene (8.1%) and β-sesquiphellandrene (7.4%). The difference in the composition can be because, during water distillation, the raw material was in contact with water, which is not the case during steam distillation. As a result, the extraction method used may affect the composition of the EO released [48]. The chemical composition of the *Z. officinale* EOs previously cited is in accordance with a number of studies, which reported the presence of these major compounds, though found in different amounts [28,39,53].

Ginger EO rich in constituents such as β-sesquiphellandrene (27.16%), caryophyllene (15.29%), α-zingiberene (13.97%), α-farnesene (10.52%) and ar-curcumene (6.62%) showed high antimicrobial and antioxidant activities [28]. *Z. officinale* EO rich in ar-curcumene (59%), β-myrcene (14%), 1,8-cineol (8%), citral (7.5%) and α-zingiberene (7.5%) exhibited high anti-inflammatory effects [54]. Ginger EO containing geranial (25.9%), α-zingiberene (9.5%), (E,E)-α-farnesene (7.6%), neral (7.4%) and ar-curcumene (6.6%) as major components was an effective antibacterial and antifungal agent, as well as a more powerful antioxidant than butylated hydroxyanisole (BHA) [27]. α-Zingiberene, a key component of ginger EO, was found in a low amount (1.64%) in the study conducted by Mesomo et al. [37]. The main components were ar-curcumene (11.32%), geranial (10.66%) followed by camphene (4.88%), β-bisabolene (4.45%) eucalyptol (3.14%), and isobornyl formate (1.95%).

Apart from the common ginger (*Z. officinalis*), many wild and cultivated species of this genus have been studied across the world and characterized for the beneficial phytochemicals present in rhizome EO [54].

The chemical composition of a species native to Thailand, *Zingiber cassumunar* Roxb., showed that major compounds were sabinene (36.71–53.50%), γ-terpinene (5.27–7.25%), terpinen-4-ol (21.85–29.96%) and (*E*)-1-(3,4-dimethoxyphenyl) butadiene (0.95–16.16%). The EO yield ranged from 1.26% to 1.37% [49]. These results are in agreement with recent results on *Z. cassumunar* rhizome EO where the major constituents were terpinen-4-ol (40.5 ± 6.6%) and sabinene (17.4 ± 1.4%) [55]. These phytochemicals have shown various pharmacological properties, including anti-inflammatory, antifungal and antibacterial effects [56]. The chemical composition of *Z. cassumunar* EO from Malysia showed that 6,9,9-tetramethyl-2,6,10-cycloundecatrien-1-one (60.77%) and α-caryophyllene (23.92%) were the most abundant components [57].

The effects of the growing conditions, different cultivation areas and harvest time were studied to compare the composition of EOs from *Zingiber montanum* Koenig. Results showed differences in their constituents according to these exogenous factors. The major components measured were sabinene (52.64–56.34%), terpinen-4-ol (7.16–10.17%) and (*E*)-1-(3,4-dimethoxyphenyl) butadiene (10.8–14.7%) [58].

Coral ginger (*Zingiber corallinum* Hance), an herbal remedy in traditional Chinese medicine, was studied for its rhizome EO. Sabinene (53.38%), α-terpinene (3.23%), γ-terpinene (2.16%), terpinen-4-ol (22.66%), β-sesquiphellandrene (1.41%) and 1,4-bis(methoxy)-triquinacene (9.64%) were the major compounds [59].

The chemical composition of *Zingiber zerumbet* (L.) Sm. var. Darcyi EO obtained by hydrodistillation from the rhizome showed that zerumbone (69.9%), α humulene (12.9%), humulene epoxide II (2.5%), caryophyllene oxide (1.1%) and camphene (1.9%) were the major constituents [60].

*Zingiber nimmonii* (J. Graham) Dalzell is an endemic species from the Western Ghats in South India. The major components of the rhizome EO were different from the rhizome EO obtained from other species. The major constituents were myrcene (5.1%), β-caryophyllene (26.9%), α-humulene (19.6%) and α-cadinol (5.2%) [61]. These results are also in accordance with Sabulal et al. [19] who showed that *Z. nimmonii* EO is a unique caryophyllene-rich natural source.

*Zingiber moran* is a local ginger variety endemic to the northeast Indian region, rich in camphene, citral, and linalool [54]. *Zingiber wrayi* var. Halabala C.K. Lim, a local herb from the Bala Forest in Narathiwat (Thailand), was investigated for its EO composition. Four compounds including *trans*-anethole (96.5%), estragol, camphor and *m*-phenylphenol [62].

## 4. The Genus *Zingiber* in Traditional Medicine

### 4.1. Medicinal Uses of Ginger

Ginger (*Z. officinale*) is an important plant with several medicinal, ethnomedicinal and nutritional values. Among different biological activities, ginger has demonstrated anti-inflammatory, antioxidant, anti-emetic, analgesic, and antimicrobial activities. Overall, they can be mainly ascribed to 6-gingerol and 6-shogaol, which represent the major compounds in ginger rhizomes, among hundreds of molecules isolated [63,64].

According to recent literature, ginger anti-inflammatory properties are mediated by the inhibition of 5-lipoxygenase or prostaglandin synthetase, which reduces biosynthesis of prostaglandins, leukotrienes and pro-inflammatory cytokines such as interleukin (IL)-1, IL-8; tumor necrosis factor (TNF)-α, and nuclear factor κB (NFκB) [63]. One clinical trial, indeed, reported its beneficial effects in reducing pro-inflammatory cytokines of patients suffering from osteoarthritis [65]. In addition, the antioxidant activity of the *Z. officinale* extract [66] has been in vitro demonstrated to inhibit the hydroxyl radicals and the lipid peroxidation products. This was consistent with further studies in animal models, which revealed as it acted by enhancing antioxidant enzyme defenses and serum glutathione [67]. Similar effects were attributed to ginger single constituents, namely, 6-gingerol, 8-gingerol, 10-gingerol, and 6-shogaol [68] as well as geranial and neral, α-zingiberene, camphene, α-farnesene, β-sesquiphellandrene [69] and zingerone [67]. 6-Shogaol, in particular, showed the most potent antioxidant and anti-inflammatory properties, due to the presence of α, β-unsaturated ketone moiety [67], while zingerone exhibited, in mice, protection against radiation-induced toxicity, increasing antiapoptotic molecules (Bcl-2) while reducing the proapoptotic ones (Bax) [70].

Together, the above reported antioxidant and anti-inflammatory properties of ginger support its preventive role against a plethora of chronic-degenerative diseases [71], including cancer, cardiovascular disorders, and diabetes.

Although still under debate, anticancer activity of ginger is, as mentioned above, mainly related to the high content of 6-gingerol and 6-shogaol. Ginger and related bioactive molecules, indeed, are effective in controlling, in vitro, viability and invasiveness of colorectal, gastric, ovarian, liver, skin, breast, and prostate cancer cells [67]. Recent evidence supports, in particular, the role of zingerone supplementation as chemopreventive agent, reducing cancer incidence in dimethyl hydrazine treated rats; the mechanism included the inhibition of cell proliferation, the induction of cell apoptosis, and the suppression of NF-κB and heme oxygenase (HO)-1 expression [70]. The proapoptotic effect and the promotion of cell cycle arrest in hepatoma and prostate cancer cells were ascribed to the activation of caspase cascade and the impairment of the nuclear translocation of NF-κB, particularly by 6-gingerol [70], which was also able to inhibit angiogenesis and invasiveness in the murine cancer models [70]. Anti-angiogenetic activity of 6-gingerol occurs by the inhibition of the vascular endothelial growth factor (VEGF), while its anti-metastatic activity could be ascribed to regulation of matrix metalloproteinases 2/9 transcription [64]. Another active compound contained in ginger is zerumbone, which induced apoptosis in pancreatic carcinoma cells, through the p53 signal pathway and increasing the activity of caspase-3 [70]. In humans, the chemopreventive effect of ginger has been mainly investigated against colorectal cancer, in virtue of its anti-inflammatory effects, similarly to those of aspirin. Ginger significantly lowered COX-1 protein expression in patients at increased risk for colorectal cancer [72], but with no effect on eicosanoid levels [73].

The role of ginger in reducing cardiovascular diseases and diabetes is highly related to its ability in controlling body weight, and reducing serum levels of glucose and lipids. Indeed, a study showed that ginger significantly decreased glucose, total cholesterol, triglycerides, free fatty acids, low density lipoproteins (LDL) and very low density lipoproteins (VLDL), whilst raised high density lipoproteins (HDL) in serum of rats with diabetic or fed with a high fat diet [67]. These effects are mainly related to zingerone [70], and less to shogaols [64]. Recently, in high fat diet fed animals, zingerone and 6-gingerol both possessed high lipolytic activity: the former by increasing the activity of norepinephrine-sensitive lipases, enhancing basal lipolysis and isoprenaline-induced lipolysis in adipocytes [64], while the latter by reducing the levels of fatty acid synthase and adipocyte-specific fatty acid binding protein [64]. In addition, 6-gingerol could prevent diabetes via the improvement of adipocyte dysfunction, since it caused the inhibition of the TNF-α mediated downregulation of adiponectin expression, as well as arachidonic acid pathway in turn inhibiting anti-platelet aggregation [64]. A clinical trial showed that ginger consumption enhanced thermogenesis and reduced feeling of hunger, suggesting a potential role in weight control [74]. In patients with type 2 diabetes, ginger improved glycemic index, total antioxidant capacity [75], insulin sensitivity and lipid profile, reducing c-reactive protein and prostaglandin E_2_ [76,77]. In peritoneal dialysis patients, for whom one of the major risk factors for cardiovascular disease is serum triglyceride concentration, the latter resulted as being reduced by daily administration of 1000 mg ginger [78].

As antimicrobial agent, ginger extract exhibited higher antifungal than antibacterial effects in vitro, showing anti-*Candida* activity against strains isolated from patients. This finding was related to the high anti-biofilm activity against *C. albicans*, at concentrations ranging from 0.625 mg/mL to 5 mg/mL [79]. Ginger was also effective against other fungal strains, such as *Fusarium* spp., and it inhibited the growth of fungi that were resistant to amphotericin B and ketoconazole [80,81,82]. Among bacteria, it showed efficacy against *Pseudomona aeruginosa*, *Staphylococcus aureus*, *Acinetobacter baumannii* [79], *Escherichia coli*, *Bacillus subtilis* and *Salmonella typhi* [83]. Furthermore, 6-gingerol and 12-gingerol showed antibacterial activity against periodontal bacteria [83], so that a clinical trial was performed to test a polyherbal mouthwash containing, among the others, the hydroalcoholic extract of *Z. officinale*; it was worth noting that it was effective in reducing gingival and plaque indices similarly to chlorhexidine mouthwash [84]. On the other hand, the antidiarrheal activity of 6-gingerol has been accredited to its ability to bind to the toxin produced by *Vibrio cholera*, rather than due to direct antibacterial activity [64].

Nausea and emesis are among the most common adverse effects of chemotherapeutics as well as frequent events during pregnancy and post-surgery anesthesia. At preclinical level, 6-gingerol showed efficacy in rats against cisplatin-induced nausea and vomiting [64]. Along these lines, a number of clinical trials and related systematic reviews and meta-analysis now support the efficacy of ginger in reducing hyperemesis during pregnancy [85,86] as well as in alleviating nausea and vomiting during chemotherapy, especially for breast cancer [87]. Similarly, ginger appeared to reduce post-anesthesia emesis in gynecological surgery [88] and after antitubercolosis drug administration [89].

Analgesic and antipyretic activities of ginger can be ascribed to 6-gingerol, as shown in rats [64]. Injection of 10 µg of 6-gingerol into the rat spinal cord was found to be effective in ameliorating neuropathic pain, via vanilloid receptor-mediated pathway [64]. In humans, ginger intake produced pain relief in primary dysmenorrhea similarly to conventional analgesic drugs [90,91], while remaining controversial in the case of ostheoartritis [92,93]. In addition, it showed an abortive effect against migraines [94], particularly when administered early and in the presence of mild migraines [95].

Further activities include gastroprotective, immunomodulatory, anti-allergy and hepatoprotective properties, in all cases mainly related to 6-gingerol [64]. In particular, ginger reduced the gastropathy induced by some drugs, such as anti-tuberculosis agents [96] and nonsteroid anti-inflammatory drugs [97].

Due to these overwhelming activities supported by preclinical evidence, people of different cultures have traditionally applied ginger as a medicinal agent for a long time ago. A vast body of anecdotal evidence, which can be used to support ginger uses and efficacy, can be found in various traditional systems of medicine belonging to Indian, Unani, Chinese, Japanese and other cultures [98].

#### 4.1.1. Ginger in the Indian System of Medicine

Ginger plays an important role in traditional Indian medicine. It is also used as an ingredient in traditional Indian drinks. Fresh ginger is one of the main spices used for making dishes, both vegetarian and non-vegetarian foods. In the Ayurveda system of medicine, ginger, either fresh or dry, has been widely used as a common household remedy for various illnesses [99,100]. Commonly, both types of ginger, which have similar properties, act as an appetizer, carminative, and stomachic [101]. Other than that, ginger is acrid, analgesic, antirheumatic, antiphlegmatic, diuretic and aphrodisiac. Ginger is also used to treat asthma, bronchitis, piles, eructation, and ascites, in order to help cleanse the throat, is useful for the voice (corrective of larynx affections), subsides vomiting, relieves flatulence and constipation, acts as a remedy for cough and relieves neck pain. In addition, the Ayurveda system also cited that ginger has anti-inflammatory and anti-edematous activities. Due to its hot property, ginger can cause dryness and, thus, is antidiarrheal [30]. Moreover, ginger is applied externally to boils and enlarged glands, and internally as a tonic in Cambodia [102]. According to Nadkarni [103], ginger also strengthens memory and removes obstruction in the vessels, incontinence of urine, and nervous diseases.

Specifically, the fresh ginger is used together with honey and ghee as a remedy for cough or alone as a remedy for acute ascites with dropsy arising from liver cirrhosis. Additionally, the juice is applied as a strong diuretic [102]. The outer skin of ginger is used as a carminative and is said to be a remedy for opacity of the cornea. On the other hand, the dry ginger has been reported to possess antiarthritic and antifilarial activities [103] while the paste of dry ginger with water is effective in recovering from fainting and is also applied externally to the eyelids. In addition, the ginger powder can also be used as a snuff. The dry ginger, in combination with dry rock salt, long pepper and black pepper is powdered and then mixed with fresh ginger juice and used as a gargle, and for the treatment of phlegmatic affections of the heart, head, neck, and chest. Moreover, the combination can also exert remarkable effects against all types of severe fevers and their associated symptoms.

Other than its uses to treat human affections, ginger is used in veterinary as a stimulant and carminative, in indigestion in horses and cattle, in spasmodic colic of horses, and to prevent gripping by purgatives [104].

#### 4.1.2. Ginger in the Chinese and Japanese Systems of Medicine

Ginger rhizome is an important drug in the Chinese and Japanese medicinal systems [98]. In Chinese medicine, fresh ginger (Rhizoma Zingiberis Recens) is used as an antiemetic, antitussive, or expectorant, and is used to induce perspiration and dispel cold, whereas the dried ginger is used for stomachache, vomiting, and diarrhea accompanied by cold extremities and faint pulse [105]. In Chinese Materia Medica, Benskey and Gamble [105] cited that ginger has the ability to: (i) release the exterior and disperse cold—used for exterior cold patterns; (ii) warm the middle burner and alleviate vomiting—used for cold in the stomach, especially when there is vomiting; (iii) disperse cold and alleviate coughing, used for coughing from acute wind, cold cough patterns, and chronic lung disorders with phlegm; (iv) reduce the poisonous effects of other herbs—used to detoxify or treat overdoses of other herbs such as *Radix Aconiti Carmichaeli Praeparata* (Fuzi) or *Rhizoma Pinelliae Ternata* (Ban Xia).

#### 4.1.3. Ginger in the Unani System of Medicine

In the Unani system, ginger is used for its anthelmintic, aphrodisiac, carminative, digestive, and sedative properties; in headaches, lumbago, nervous diseases, pains, and rheumatism; and for strengthening of memory [103]. Ginger is also used in veterinary medicine in horses and cattle for rheumatic complaints, as an antispasmodic and a carminative in atonic indigestion [106,107].

### 4.2. Examples of Ginger Species and Their Uses in Traditional Medicine

Ginger has been used as a traditional medicine since ancient times. It is considered a medicinal plant as it has several curative properties in treating different diseases. In the following sections, some ginger species known for their medicinal properties are reported.

#### 4.2.1. *Zingiber officinale* Roscoe

*Z. officinale* is the best known *Zingiber* plant in the ginger family and is also referred to as garden ginger or common ginger. This ginger is used in Ayurveda and Chinese medicine, as previously mentioned, in both fresh and dried preparations, for curing heart problems, treating stomach upset, diarrhea, headaches, and nausea. Other than that, *Z. officinale* has also been used to promote the release of bile from the gall bladder, reduce joint pain from arthritis, treat heart and lung diseases; relieve cough and cold, throat infection and even the removal of warts and corns. In both the Chinese and Japanese systems of medicine, fresh ginger is used for its warming properties and as a remedy for coughs and nausea, whereas dried ginger is indicated for ailments of the digestive system. In aromatherapy, the essential oil of ginger is used for muscle and joint pain, sprains, colds, nausea, diarrhoea, alcoholism and helping the healing of broken bones [108,109,110]. The rhizome of *Z. officinale* can also be prepared as a tea for indigestion and increasing the blood circulation and sense of well-being [111]. In addition, the rhizome extracts have been used in Malay traditional culture to treat various types of ailments such as inflammatory- and pain-mediated diseases, worm infestation, and diarrhea.

#### 4.2.2. *Zingiber montanum* (J. Koenig) Link ex A. Dietr

The rhizomes of *Z. montanum*, also known as “cassumunar ginger”, are used throughout tropical Asia primarily as a carminative and stimulant for the stomach, and against diarrhea and colic [112,113]. The pounded rhizome is traditionally used in Indonesia as a poultice against headache while the Malaysians used the rhizome internally as a vermifuge and for postpartum medication. Moreover, in Laos, the rhizome is applied against abscesses, fever, colic, diarrhea and other intestinal disorders, as a depurative, as well as a poison antidote, whereas, in Thailand, the rhizomes are taken against asthma and used as the main ingredient in massage oil to relieve muscle pain [112,114]. Other than that, the rhizome paste is consumed orally by the people living in northeast India to treat dyspepsia and stomach bloating [113,114].

#### 4.2.3. *Zingiber mioga* (Thunb.) Roscoe

Also known as Japanese ginger or myoga ginger, this perennial herb is native to Japan, China and the southern part of Korea. The subterranean stem and young flower buds of this species can be used to cure menstrual irregularity, leucorrhea, heart disease, and eye inflammation. It can also be used as an expectorant. In addition, *Z. mioga* is used to treat cough and rheumatism in China and consumed throughout Japan to relieve insect bites, eye inflammation, cough and rheumatism [115,116].

#### 4.2.4. *Zingiber spectabile* Griff.

Native to maritime Southeast Asia such as peninsular Malaysia and peninsular Thailand, it is a species of ginger commonly known in the west as “beehive ginger”. Different parts of *Z. spectabile* are widely used in the Malay traditional medicine to treat various ailments. For example, the pounded leaves are used as poultice to treat swelling or applied topically to the required part of the body to treat burns, backaches, headaches and back pain [116]. The juice from leaves has been used by the Temuan tribe, which is one of the indigenous populations found in the Ayer Hitam Forest, Perak, Malaysia, to treat eye infections and to soothe swollen eyes [117]. In Thailand, the fresh pounded leaf infusion can be used to wash the infected eyelids or treat inflammation of the eye [118]. Moreover, the paste of *Z. spectabile* fresh leaves is patched onto open wounds to heal them, while the water obtained from boiling the leaves is said to be effective against sinus ailments, and is used as a facial and nasal wash. The rhizomes are used in the treatments of cancer, cough and asthma, as a stimulant, tonic and germicide. *Z. spectabile* is also used in recipes for medicinal bath by mothers after giving birth and during the maternity period, especially for post-natal bath by boiling the whole plant [119].

#### 4.2.5. *Zingiber zerumbet* (L.) Sm.

*Z. zerumbet*, also known as shampoo ginger and native to India, is found in many tropical countries. The rhizome extracts of this species have been used to treat a diverse range of ailments. For example, the Hawaiians applied the fresh pounded rhizome as medicine for indigestion and other ailments. Traditionally, the ground rhizomes, mixed with a ripe noni (*Morinda citrifolia* L.) fruit, can be used to treat severe sprains while the pulp, placed in a cloth, can be loosely bound around the injured area. The cooked and softened rhizome can also be used to treat toothache or caries by pressing it into the hollow and left for as long as was needed, while the ground and strained rhizome material is mixed with water and drunk to treat a stomachache [120].

Other than that, the rhizome of *Z. zerumbet* has been generally cited to be used in the treatment of inflammation, diarrhea, stomach cramps, bacterial infections, fever, flatulence, allergies and poisoning. The powdered rhizomes are used in the treatment of ear infections and toothache, while the tea of *Z. zerumbet* rhizome is used to treat stomach disease. In addition, the juice of cooked rhizome can be used in the treatment of worm infection in children. The leaves are also used in therapies for joint pain [121].

#### 4.2.6. *Zingiber ottensii* Valeton

In Indonesia, the stem is traditionally used as part of a sedative lotion by the Javanese people, while, in Sumatra, the stem is used as potherb for postpartum care. On the other hand, the traditional midwives in Perak, Malaysia used the rhizomes and leaves as a poultice applied on the body of the women in confinement. In addition, the leaves are used as a poultice for lumbago [122]. The pungent rhizomes are pounded into a poultice and used by women after childbirth, or are added to a mixture to make a sedative lotion or a tonic [123].

## 5. Essential Oil Obtained from Genus *Zingiber* Plants as a Food Preservative

Consumers are increasingly concerned with the use of chemical agents in foods, due to safety issues. The consumer today values products that have “natural” preservatives and a “clean label” or products with no artificial ingredients. Traditional food antimicrobials are proven and remain extremely effective in achieving shelf life and food safety goals, and, when they are evaluated under real food processing/handling conditions, antimicrobial resistance does not appear to be a major phenomenon [124].

A wealth of new technologies including high hydrostatic pressure, ionization radiation, and bioactive packaging can reduce or eliminate the need for utilization of traditional food preservatives. The major drawback of these technologies is associated with cost and feasibility of use in a range of food products. In the past decade, attention has been focused on the utilization of plant essential oils as natural antimicrobials in foods. Essential oils have been found to exhibit a broad range of activity against spoilage and pathogenic bacteria associated with food. They have been derived, in particular, from *Thymus vulgaris* (thyme), *Origanum majorana* (marjoram), *Origanum vulgare* (oregano), *Ocimum basilicum* (basil), *Cymbopogon citratus* (lemon grass), and *Caryophyllus aromaticus* (clove) have been evaluated for the ability to control or inactivate various foodborne pathogens in vitro or in select foods [125]. A key advantage to the use of essential oils from those plants is that they are considered generally recognized as safe (GRAS) for use in food.

As previously introduced, ginger is an important spice used throughout Asia and has gained considerable global popularity as an ingredient in food due to its unique flavor. The flavor is derived from both volatile and non-volatile compounds (gingerol, shogaol, and zingiberene), while the antimicrobial activity is due to the presence of essential oils, such as camphene, linalool, α-pinene, and borneol. They have been reported effective against *Aeromonas hydrophila*, *Bacillus subtilis*, *Bacillus cereus*, *Listeria monocytogenes*, *Salinococcus roseus*, *Halococcus turkmenicus*, *Halococcus morrhuae*, *Pseudomonas aeruginosa*, *Staphylococcus aureus*, *Salmonella* spp., *Escherichia coli*, *Vibrio cholera*, *Vibrio paraheamolyticus*, *Aspergillus niger*, *Mucor* spp., *Candida albicans*, and *Penicillium* spp. [8,125,126,127,128]. In particular, the part of the plant (leaf or rhizome) selected and method of extraction used can have a profound impact on the antimicrobial activity [37,129]. Ginger essential oils extracted from both the leaf and rhizome exhibited antibacterial activity against *B. licheniformis*, *B. spizizenii*, *S. aureus*, *E. coli*, *K. pneumoniae*, and *P. stutzeri* (minimum inhibitory concentration, MIC = 0.16–0.63 mg/mL) [22]. The leaf oil was composed mainly by β-caryophyllene, the rhizome oil contained mostly monoterpenoid, in particular camphene and geranial [22]. In general, activity was higher against the Gram-positive bacteria, consistently with others’ studies [24,130,131]. Moreover, the antibacterial activity of ginger essential oil is comparable or even higher than that of essential oils derived from other plants. A comparison of ginger, eucalyptus, and sweet orange essential oils found that ginger essential oil exhibited the highest antibacterial activity [85]. However, *V. parahaemolyticus* was resistant to all of the essential oils evaluated. Ginger, thyme, coriander, marjoram, mustard, chamomile, licorice and nigella essential oils were evaluated for their activity against Gram-negative and Gram-positive foodborne pathogens [132]. Ginger, thyme, and coriander showed the highest antibacterial inhibition against the strains of bacteria tested.

As antimould agent in food preservation, zingiber essential oils showed controversial properties. If yeasts in food spoilage present no human health concern, moulds can produce mycotoxins, which, in the worst-case scenario, can result in chronic toxicity and death. Essential oils of ginger exhibited poor activity against *Penicillium* spp. (MIC = 869.2 mg/mL) and no activity against *A. niger* [24]. These results are in agreement with other reports [86]. In contrast, ginger essential oil was reported to inhibit the growth of *Fusarium verticilliodes* (MIC = 2500 μg/mL) and the production of fumonisin B1 and fumonisin B2 at concentrations of 4000 and 2000 μg/mL, respectively [88]. Eventually, the growth of yeasts, *Candida tropicalis* and *Candida utilis* was not inhibited by exposure to ginger essential oil [131].

The potential use of ginger essential oil as food preservatives and, therefore, could include the control of microorganisms’ overgrowth in fresh and fresh-cut fruits and vegetables that are intended to be consumed without a cooking step. As an example, shredded green or unripe papaya is used in several types of Asian cuisine, including Thai papaya salad. The ability of three amounts of ginger essential oil (5, 10 or 15 µL) to control bacterial, mould, and yeast growth in shredded green papaya stored at 13 °C for four days was evaluated [128]. The populations of bacteria and yeasts were approximately 3-log lower on shredded green papaya treated with the highest concentration of ginger essential oil compared to the untreated control [128] A further application could be to reduce the risk of contamination in raw poultry. The latter can be contaminated with *Salmonella*, *Campylobacter*, or both. Often, poultry carcasses are rinsed or immersed in a water bath containing an antimicrobial chemical (e.g., chlorine). The practice is used in part to reduce the microbial load, including foodborne pathogens, on the carcass prior to packaging and shipment to the consumer. Avoiding toxic compounds, consumers would likely meet utilization of essential oils to control microorganisms on poultry carcasses with a positive response. In a study, chicken breast and whole leg samples were immersed for 2 min in various concentrations of ginger essential oil (1:150, 1:250 and 1:550) and then total aerobic bacteria, *E. coli*, *S. aureus* and *Salmonella* populations determined [89]. A highly significant reduction in population of total aerobic bacteria—*E. coli*, *S. aureus* and *Salmonella*—occurred following treatment, higher than a 3-log reduction in the populations of *E. coli* and *Salmonella* on the surface of chicken was achieved [89].

Concerning safety issues, ginger is included among spices provided from natural sources, defined as GRAS by the American Food and Drug Administration (FDA) [133]. From clinical trials, we can remark about which concentrations can be considered of reasonably safe for human consumption. The daily dose of oral administration of ginger ranged from 500 mg/day to 1000 mg/day; at these concentrations, adverse effects were rare and mainly in the form of gastrointestinal discomfort [85,92]. Considering ginger metabolites (6-, 8- and 10-gingerols and 6-shogaols), a clinical trial on healthy volunteers showed no toxicity up to 2000 mg [134]. At a preclinical level, using the rat animal model, the acute oral lethal dose of ginger essential oil was found to be over 5 g/kg of weight, and certain teratogenicity, embryonic loss and mutagenicity could not be definitely ruled out [135,136].

Altogether, these findings suggest that ginger essential oils are active against microorganisms, mainly bacteria, as demonstrated predominantly through in vitro studies. Overall, a dearth of literature on ginger as food preservative exists, even more if compared with the plethora of investigations about essential oils derived from other plants [8,125].

The research, therefore, is encouraging, but many more studies should be completed to evaluate essential oils of ginger in or on complex foods, before solid conclusions can be drawn in support of their practical applications. These studies should also evaluate organoleptic changes of food including impact on taste, odor, and color. A key factor in the use of ginger essential oil as an antimicrobial in food or food processing might be cost. However, despite the cost, consumer acceptance of the ginger essential oils, as a natural alternative to traditional food preservatives, is expected to be embraced with enthusiasm, in the perspective of reducing the need of chemical agents.

## 6. Conclusions

Indian, Chinese, Japanese, and Unani systems of medicine use ginger extracts to deal with pain and inflammatory disease since decades. At the end of this survey, members of the genus *Zingiber* represent a promising and innovative source of natural bioactive agents, mainly gingerols, shogaols and zingerone.

Clinical evidence suggests their efficacy, in particular, in managing hyperemesis related to pregnancy or chemotherapy. As antimicrobial agents, ginger essential oils appear especially effective in the management of food contaminations, increasing the shelf life of food. Given their reasonable safety data, members of the genus *Zingiber* may constitute a valid alternative towards common drugs to manage nausea and vomiting, and towards chemical food preservatives. The latter issue meets the increasing concern of consumers aware of the potential health risks associated with the conventional antimicrobials in food.

## Figures and Tables

**Table 1:** Extraction methods, major constituents and biological activities of *Zingiber* spp. essential oils.

Plant	Extraction Methods	Major Compounds	Biological Activities	References
*Z. officinale*	Hydrodistillation	ar-curcumene (11.32%), geranial (10.66%), camphene (4.88%), eucalyptol (3.14%), isobornyl formate (1.95%), α-zingiberene (1.64%)	Antibacterial	[37]
*Z. officinale*	Hydrodistillation, microwave assisted hydrodistillation, solvent-free microwave hydrodistillation, improved solvent-free microwave extraction	α-zingiberene (17.4–25.4%), ar-curcumene (14.1–16.4%), β-bisabolene (9.9–12.5%), β-sesquiphellandrene (9.7–13.4%)	NR *	[46]
*Z. officinale*	Hydrodistillation	Geranial (25.9%), α-zingiberene (9.5%), (*E*,*E*)-α-farnesene (7.6%), neral (7.4%), ar-curcumene (6.6%)	Antibacterial, antifungal, antioxidant	[28]
Three sub-Himalayan *Z. officinale* cultivars (Gorubathane, Shingboi Thingria	Hydrodistillation	Gorubathane: α-zingiberene (32.2%), β-sesquiphellandrene (10.9%); Thingria: α-zingibirene (12.58%), ar-curcumene (9.89%); Shingboi: geranial (20.07%), neral (9.44%)	NR	[44]
Fresh and dry *Z. officinale* var. Nedumangadu	Hydrodistillation	Fresh ginger: α-zingiberene (28.6%), geranial (8.5%) ar-curcumene (5.6%), β-bisabolene (5.8%); Dry ginger: α-zingiberene (30.9%), ar-urcumene (11%), β-bisabolene (7.2%), β-sesquiphellandrene (6.6%), germacrene-D (4.2%)	Antibacterial, antifungal	[51]
*Z. officinale*	Hydrodistillation	α-zingiberene (28.62%), camphene (9.32%), ar-curcumene (9.09%), β-phellandrene (7.97%)	Antifungal, antioxidant	[45]
*Z. officinale*	Hydrodistillation	β-sesquiphellandrene (27.16%), caryophyllene (15.29%), zingiberene (13.97%), α-farnesene (10.52%), ar-curcumin (6.62%)	Antibacterial, antioxidant	[28]
*Z. montanum*	Hydrodistillation	Sabinene (52.64–56.34%), terpinen-4-ol (7.1–10.17%), (*E*)-1-(3-4-dimethoxyphenyl) butadiene (10.8–14.7%)	NR	[58]
*Z. cassumunar* (three native cultivars)	Hydrodistillation	Sabinene (36.71–53.50%), γ-terpinene (5.27–7.25%), terpinen-4-ol (21.8–29.96%), (*E*)-1-(3-4-dimethoxyphenyl) butadiene (0.95–16.16%)	NR	[49]
*Z. cassumunar*	Steam distillation	6,9,9-tetramethyl-2,6,10-cycloundecatrien-1-one (60.77%), α-caryophyllene (23.92%)	Slight antimicrobial	[57]
*Z. officinale*	Steam distillation	ar-curcumene (59%), b-myrcene (14%), 1,8-cineol (8%), citral (7.5%), and α-zingiberene (7.5%)	anti-inflammatory	[39]
*Z. zerumbet* var. Darcyi	Hydrodistillation	zerumbone (69.9%), α-humulene (12.9%), humulene epoxide II (2.5%), caryophyllene oxide (1.1%), camphene (1.9%)	NR	[60]
*Z. corallinum*	Steam distillation	Sabinene (53.38%), ɑ-terpinene (3.23%), γ-terpinene (2.16%), terpinen-4-ol (22.66%), β-sesquiphellandrene (1.41%), 1,4-bis(methoxy) triquinacene (9.64%)	NR	[59]
*Z. nimmonii*	Hydrodistillation	Myrcene (5.1%), β-caryophyllene (26.9%), α-humulene (19.6%), α-cadinol (5.2%)	Larvicidal and repellent	[31]
*Z. nimmonii*	Hydrodistillation	β-caryophyllene (42.2%), α-humulene, α-caryophyllene (27.7%)	Antimicrobial	[19]
*Z. moran*	Hydrodistillation	Camphene, citral, linalool	Cytotoxic	[54]
*Z. wrayi* var. Halabala	Steam distillation	*trans*-anethole (96.5%)	Antibacterial	[62]

* NR, not reported.
